# Programing stimuli-responsiveness of gelatin with electron beams: basic effects and development of a hydration-controlled biocompatible demonstrator

**DOI:** 10.1038/s41598-017-17734-y

**Published:** 2017-12-12

**Authors:** Stefanie Riedel, Benedikt Heyart, Katharina S. Apel, Stefan G. Mayr

**Affiliations:** 1 Leibniz Institute of Surface Engineering (IOM), Leipzig, 04318 Germany; 2 Division of Surface Physics, Department of Physics and Earth Sciences, University of Leipzig, Leipzig, 04103 Germany

## Abstract

Biomimetic materials with programmable stimuli responsiveness constitute a highly attractive material class for building bioactuators, sensors and active control elements in future biomedical applications. With this background, we demonstrate how energetic electron beams can be utilized to construct tailored stimuli responsive actuators for biomedical applications. Composed of collagen-derived gelatin, they reveal a mechanical response to hydration and changes in pH-value and ion concentration, while maintaining their excellent biocompatibility and biodegradability. While this is explicitly demonstrated by systematic characterizing an electron-beam synthesized gelatin-based actuator of cantilever geometry, the underlying materials processes are also discussed, based on the fundamental physical and chemical principles. When applied within classical electron beam lithography systems, these findings pave the way for a novel class of highly versatile integrated bioactuators from micro- to macroscales.

## Introduction

Stimuli responsive biological systems are omnipresent in nature and can be regarded one of the ingredients of life. Contemporary material developments for biomedical applications, e.g. in the fields of implantology and tissue engineering, increasingly follow this paradigm by focusing on smart and adaptive, biomimetic and biodegradable assemblies in replacement for traditionally passive approaches. When heading for active elements in regenerative medicine or drug delivery, control of switching behavior to environmental conditions constitutes an integral challenge, in addition to their programmable bioabsorbability. Thereby, polymeric materials, especially synthetic materials^1^, are already investigated in order to construct active elements such as actuating objects and self-expanding stents^2,3^. Furthermore, the actuatoric potential of biological systems found in nature were investigated in more detail^4^. However, bio-polymers, especially collagenous materials such as gelatin, have a similarly high potential for applications as active elements but are not yet well studied. As we will demonstrate in the following, simple biologically-derived gelatin that has been appropriately modified with energetic electron beams constitutes a highly promising candidate to fulfil these specifications towards active elements e.g. switchable stents and scaffolds or active control elements of certain environmental parameters such as in microfluidic systems.

Gelatin is derived from the natural hydrogel collagen. Due to its biodegradability and biocompatibility, gelatin represents an important material for biomedical applications *in vitro* and *in vivo* ^5,6^. A disadvantage of unmodified gelatin is its low gel-sol-transition temperature *T*_*GST*_. Even though *T*_*GST*_ varies for different gelatin types, for all of them it lies far beneath the human body temperature^7^. By modifications such as crosslinking, *T*_*GST*_ can be increased and gelatin can be adapted to become usable for applications at human body temperature^8^. As some crosslinking methods have been reported to reveal cytotoxic effects (e.g. glutaraldehyde^9^), reagent-free methods are highly desirable in the areas of biomedical and pharmaceutical applications. High energy electron irradiation represents such a nontoxic crosslinking method for gelatin modifying its properties, such as thermal stability, structure and swelling while maintaining chemical structure^10^ and biocompatibility^11^. In addition, even electron doses of just a few kGy are known to sterilize gelatin^12^, which makes it attractive for biomedical applications. During irradiation, high energetic electrons break bonds within the polymeric chains due to homolytic scission, e.g. of C-H bonds, which causes the formation of radicals^13^. Additionally, macro radicals form due to radiolysis of water molecules and further attack the polymer chains causing formation of more radicals which recombine into crosslinking covalent bonds within the chains.

With the vision of a novel class of highly versatile, optionally biodegradable actuators for medical applications, the present work thus employs electron beams to modify, reassemble and functionalize bio-derived gelatin for tailored stimuli responsiveness in a physiological environment with response to hydration and changes in pH-value as well as ion concentration. Within this scope, the phenomenology of swelling, which is solvent diffusion into the gelatin matrix, and its modification by electron beam treatment constitutes the central aspect. While swelling, thermodynamic equilibrium between solvent and gel is reached, if the chemical potential of the solvent in- and outside the gel is the same ${\mu }_{s}^{in}={\mu }_{s}^{out}$, which can be recast into the requirement that the total osmotic pressure *π* vanishes. Due to Flory and Rehner, the latter is composed of three contributions *π* = *π*_*el*_ + *π*_*mix*_ + *π*_*ion*_ stemming from the elastic forces counteracting expansion, solvent-gel mixing and ion-solvent electrostatic effects, respectively. Following a “design by understanding” approach, we first focused on a thorough quantitative assessment of the phenomenology of swelling behavior in electron-beam-modified gelatin as function of electron dose, gel concentration, pH-value as well as ion concentration. The resulting parameter-property-relationships established from these studies was then the basis for construction of custom actuators, as we demonstrate with a hydration-sensitive gelatin cantilever. They might have an application perspective in self-expanding stents.

## Results

### Development of an active gelatin element

Active elements that deform in a programmable manner upon exposure to body fluids constitute a very versatile class of actuators that could prove useful in numerous applications in biomedicine. Among them, a bilayered cantilever geometry, that reveals a macroscopic curvature change Δ*κ* due to different expansion Δ*ε* within the individual layers poses a particularly attractive prototype. Due to a well-defined structure, it reveals a theoretically well-predictable response given by^14^

1 $${\rm{\Delta }}\kappa =\frac{6{B}_{1}{B}_{2}({h}_{1}+{h}_{2}){h}_{1}{h}_{2}{\rm{\Delta }}\varepsilon }{{B}_{1}^{2}{h}_{1}^{4}+4{B}_{1}{B}_{2}{h}_{1}^{3}{h}_{2}+6{B}_{1}{B}_{2}{h}_{1}^{2}{h}_{2}^{2}+4{B}_{1}{B}_{2}{h}_{2}^{3}{h}_{1}+{B}_{2}^{2}{h}_{2}^{4}}$$

where *B*_*i*_ = *E*_*i*_/(1 − *v*_*i*_) and *h*_*i*_ denote the biaxial moduli and thicknesses, respectively of the layers *i* = 1,2 with Young’s moduli *E*_*i*_ and Poisson ratios *v*_*i*_. Despite being as simple as this, it could already constitute the basis of a stent demonstrator that changes its perimeter upon hydration.

In view of equation (1), maximum curvatures are expected, if sufficiently thin layers are combined in an appropriate reference state to yield a large difference in strain upon hydration. Systematic swelling studies of electron irradiated gelatin (shown in detail within the Supplementary Figs S1 and S2) indicate reduced swelling of high irradiation doses as well as low gel concentrations. Consequently, by combining two layers with low concentration and high dose as well as high concentration and low dose, a response to hydration by significant deformation is expected.

**Figure 1:**
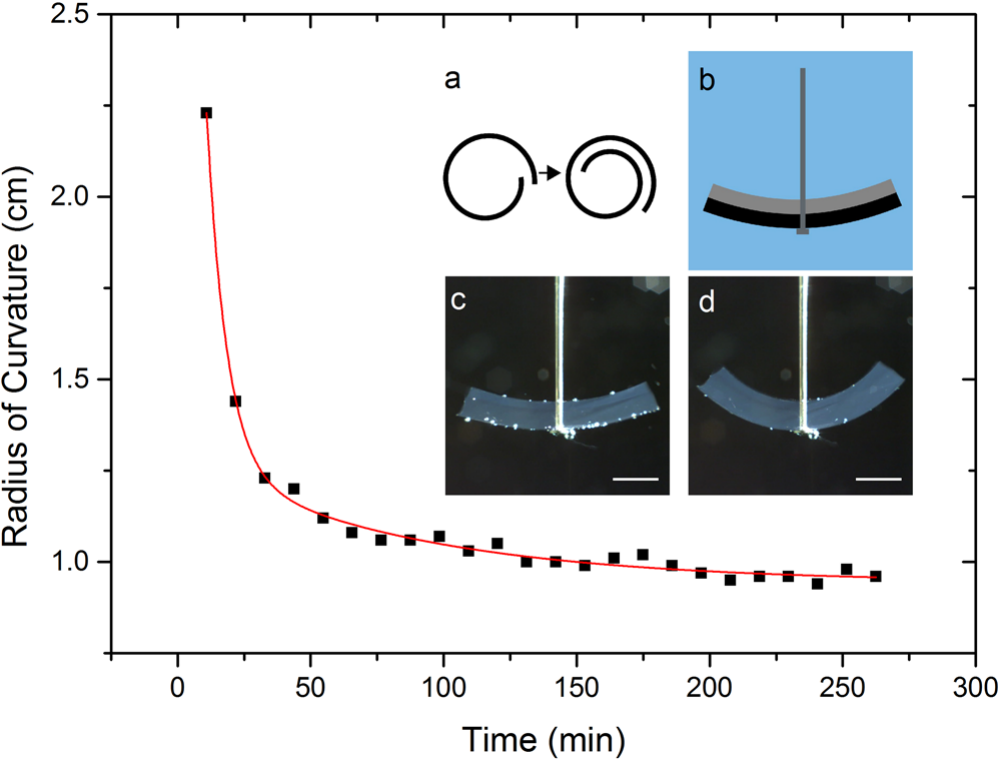
Time resolved bending of a water-sensing gelatin bilayer. The top layer consists of 4 wt% gelatin irradiated with 40 kGy. The bottom layer consists of 10 wt% gelatin irradiated with 5 kGy. The red line represents a two-phase exponential decay function. Inset: (**a**) Scheme of a stent demonstrator, (**b**) schematic drawing, (**c**) gelatin bilayer in water before deformation and (**d**) gelatin bilayer in water after deformation. Scale bars indicate 5 mm.

As a seminal example, we started with a layer of 4 wt% gelatin with a thickness of approx. 1.5 mm, that was irradiated with a total dose of 35 kGy after polymerization. Subsequently, a second layer of 10 wt% gelatin with the same thickness was deposited on top of the first layer, again polymerized and electron beam treated with a dose of 5 kGy leading to a final dose of 40 kGy and 5 kGy for the 4 wt% and the 10 wt% layer, respectively, as schematically shown in Fig. 1b. Deformation of the gelatin bilayers upon exposition to deionized water at room temperature was recorded and evaluated (as shown in Fig. 1), yielding curvature changes and deformation times of more than 100% and several hours, respectively. A movie showing the deformation of the bending cantilever can be found within the Supplementary Video S3).

### Programing stimuli responsiveness

**Figure 2:**
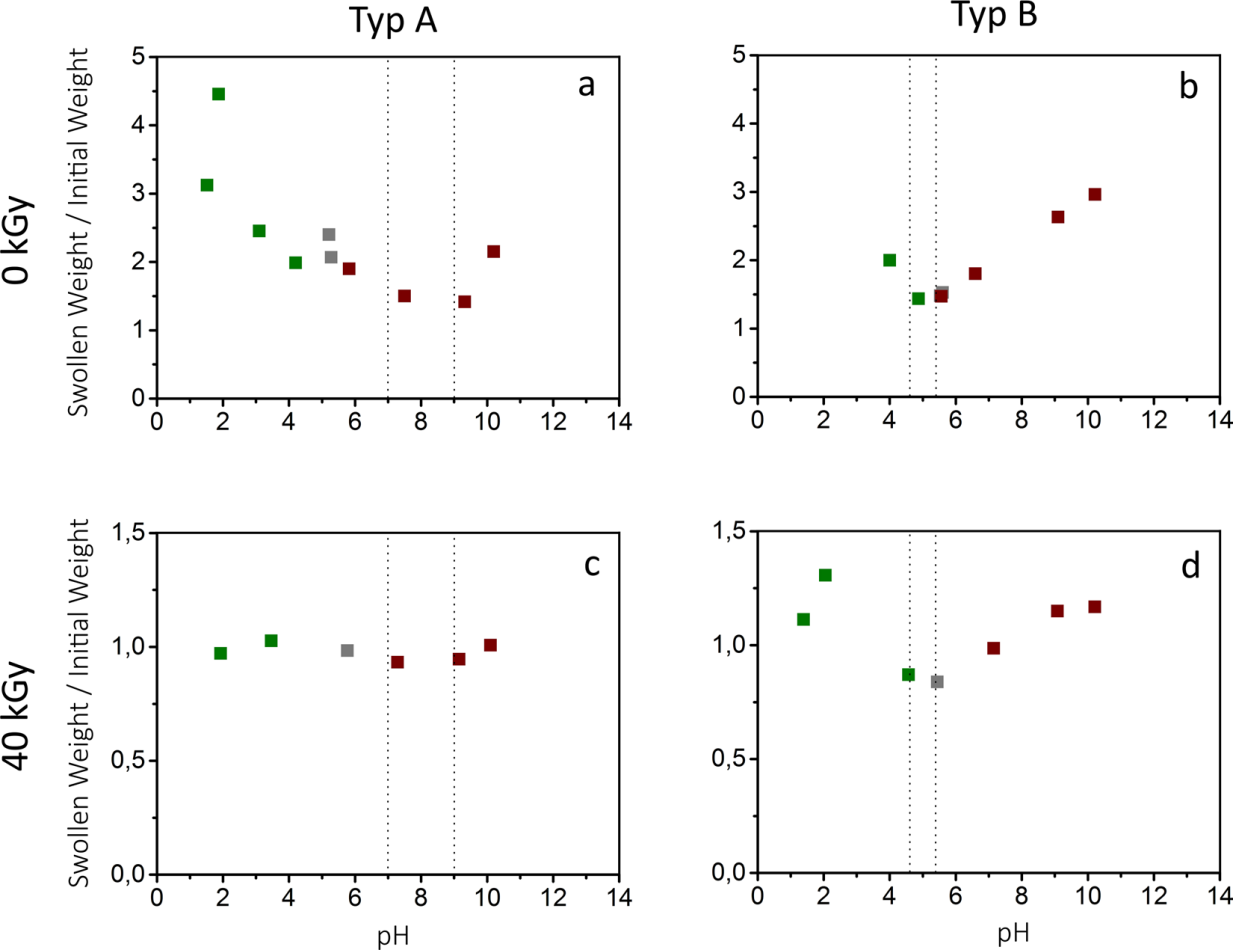
Ratio of swollen weight to initial weight depending on pH-value of unirradiated 10 wt% gelatin (**a** and **b**) and samples irradiated with 40 kGy (**c** and **d**). Two gelatin types, type A (**a** and **c**) and type B (**b** and **d**) were investigated. Swollen weights were determined after 24 h. The dashed lines indicate the range of the IEP. The pH-value was reached by using H_2_SO_4_ (green), deionized water (grey) and NaOH (red).

In view of potential biomedical applications, stimuli responsiveness of electron irradiated gelatin to changes in pH-value as well as salt ion concentration is highly relevant due to their presence and as an alternate method for switching. Therefore, swelling of irradiated and unirradiated gelatin depending on the pH-value ranging from pH 2 to pH 11 (see Fig. 2) is evaluated. By comparing different gelatin types (type A and B), the influence of the isoelectric point (IEP) on swelling is studied. The results show a strong dependence of swelling on the pH-value which seems to be correlated to the IEP, where swelling shows a minimum.

**Figure 3:**
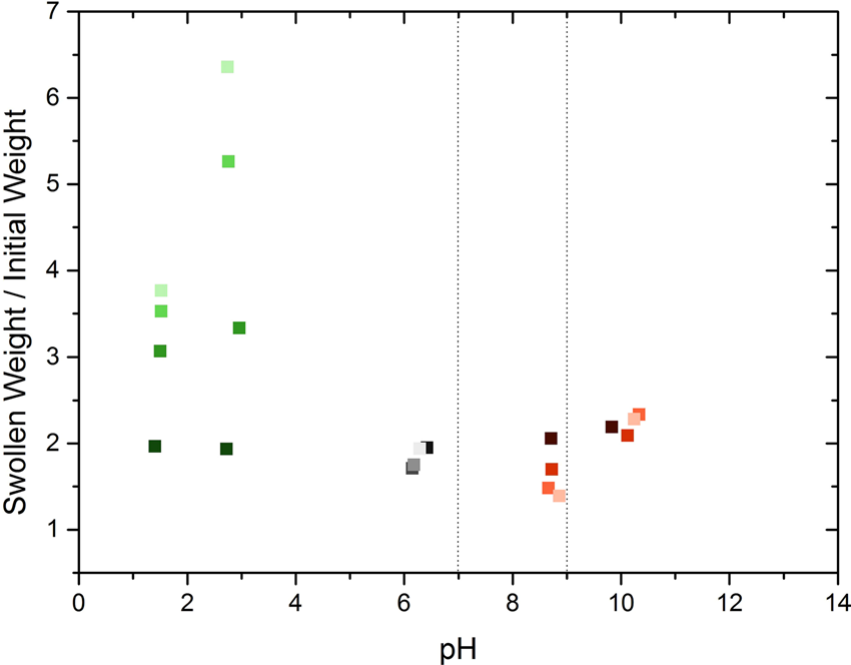
Ratio of swollen weight to initial weight of 10 wt% type A gelatin after 24 h in solution with different pH-values and NaCl concentrations. The pH-values were adjusted by H_2_SO_4_ (green), deionized water (grey) and NaOH (red). The NaCl concentrations are represented by different brightness from light to dark for 0, 0.005, 0.05 and 0.5 M. The dashed lines indicate the range of the IEP.

The impact of varying salt concentrations was analyzed in respect of NaCl as an important salt in living organisms (see Fig. 3). Thereby, NaCl concentrations between 0 M and 0.5 M were studied for similar pH-ranges as before. It is shown that an increased concentration of NaCl affects gelatin towards a decreased swelling. However, the influence of the salt is minimized close to the IEP.

## Discussion

We demonstrated that it is possible to build an active element from electron irradiated gelatin by using a bilayered system responding to hydration by deformation. Although the performance characteristics are convincing on basic grounds and might be already suitable for biomedical applications, they have a high potential for optimization, particularly in terms of time scale, magnitude and stimuli responsiveness. While we will devote the rest of our manuscript to the latter two, the former is expected to be physically governed by the time required for water to diffuse throughout the hydrogel layers. Supposed that the diffusion speed of water within the hydrogel is a material’s constant, it is expected that modification of the surface to volume ratio is the method of choice to increase cantilever response speed, that can be realized by going for smaller thicknesses of the bilayers or applying porous gelatin^15^.

Programing a desired shape change upon hydration can be realized by tuning the system’s synthesis conditions. The data obtained within the present (see Fig. 3 and Supporting information Figs S1 and S2) and our previous studies^10^ regarding the dependence of gelatin swelling on gel concentration, irradiation dose, pH-value and salt concentration constitute a sufficiently large database of synthesis parameters. Sticking exactly with the cantilever geometry realized above, differences in swelling ratios of more than 500%^10^ promise radii of curvatures below 1 mm upon hydration. In view of equation (1), further improvement of the deformation can be achieved by a decrease of layer thickness as well as elastic modulus. While the former can be easily realized, the latter one is restricted by the system stability. An increase in elastic modulus can be achieved by decreasing gel concentration and irradiation doses. However, both adaptions lead to loss in stability and thereby functionality of the system. This has to be taken into account when developing such a bilayered system. Furthermore, larger deformation effects are certainly possible by employing a lever-type of geometry, e.g. by extending the ends of the cantilever with “passive” material, while keeping the cantilever as active element. This constitutes an approach of mechanical amplification, that is quite common in the field of more traditional “shape changing materials”^16^.

Our investigations of swelling dependency on the pH-value indicate a strong dependence on the pH-value for both types of unirradiated gelatin (Fig. 2a and b) and irradiated gelatin type B (Fig. 2d). Close to the IEP, swelling is decreased while it increases with increasing distance to the IEP. These results correspond to observations from Yang *et al*.^17^. The swelling minimum at the IEP is explained by a lack of ion-solvent electrostatic effects. The gelatin polymer has no net charge and therefore, no osmotic pressure is generated by electric charge differences^18^. In view of equation (1), the term *π*_*ion*_ plays no role at the IEP and swelling just depends on *π*_*el*_ and *π*_*mix*_, i.e. gelatin concentration and degree of crosslinking. However, with increasing distance from the IEP, osmotic pressure is generated by differences in electric charges and thus swelling increases as seen in the experiments. However, there is no significant dependency of swelling of irradiated gelatin type A on the pH-value (Fig. 2c). As a result of electron irradiation, swelling is decreased (as shown in Supplementary Fig. S2 and earlier studies^10^) so that the influence of the pH-value vanishes and no pH dependence can be observed.

Our investigations further show stronger swelling at pH 2–3 (Fig. 2a and d). At this pH-value, gelatin has its dissociation maximum, i.e. there is a maximum of free ions in the gel. Regarding to the contributions to the osmotic pressure, the influence of *π*_*ion*_ maximizes which explains the observed increase in swelling. By decreasing the pH-value beyond the dissociation maximum at pH 2–3, the conjugated base of the added acid combines with the protein ions into a protein salt. Thereby, the amount of free ions is reduced which reduces the osmotic pressure and the swelling decreases again (Fig. 2a and d). However, no peak is observed for unirradiated type B gelatin (Fig. 2b) and for irradiated type A gelatin (Fig. 2c) caused by dissolution of the samples and the strong decrease in swelling, respectively. A similar effect as for small pH-values is observed at higher pH values than the IEP (Fig. 2a,b and d). At a basic pH-value, the gelatin is negatively charged and the osmotic pressure increases with increasing pH-value due to the Donnan effect. Similar to small pH values, gelatin has also a dissociation maximum at high pH-values. However, no swelling maximum (as in Fig. 2a and d) at high pH values could be observed for all measurements as it was impossible to reach higher pH values due of the amphoteric character of gelatin. The gelatin buffers the basic solution to pH values under pH 11, so no higher pH-values can be reached.

The salt depending swelling measurements indicate a large impact of salt such as NaCl on swelling of crosslinked gelatin. Close to the IEP, high concentrations of NaCl slightly support swelling while low concentrations decrease swelling. This effect is explained by the larger amount of salt ions diffusing into the polymer network and transporting additional water molecules into the gel. In contrast, close to the dissociation maximum of gelatin around pH 2–3, the opposite is observed; high NaCl concentrations hinder swelling while low concentrations support swelling. At the dissociation maximum, the high amount of protein ions creates high osmotic pressure increasing swelling ability. However, by adding large amounts of salt, the free salt ions reduce the impact of the protein ions on the osmotic pressure and so swelling is reduced. The results further show a pH-independent and constant ratio of swollen to initial weight of approx. 2 for all gels treated with 0.5 M NaCl indicating that with increasing NaCl concentration gelatin swelling becomes more and more independent from the pH-value and thus the degree of dissociation.

Concerning applications of such a bilayer element at physiological conditions, the response at a pH of 6–8 and a NaCl concentration of 0.15 M has to be considered. At the relevant pH-range, type A and type B gelatin show the lowest differences in swelling between unirradiated and irradiated samples. However, the difference is sufficient to obtain deformations as demonstrated before (see Fig. 1 and Supplementary Video S3). Adaption of the response can be improved by adaption of geometry and element structure. At physiological NaCl concentrations, swelling of unirradiated type A gelatin is slightly increased compared to the absence of NaCl. This would lead to an even higher deformation-response of the system.

The obtained results indicate that, as an option for *ex vivo* applications, the response can be additionally tuned by adaption of pH and salt concentration beyond physiological conditions leading to stronger deformation.

## Conclusion

In conclusion, we demonstrated how to build a highly promising actuating bilayer system composed of the biopolymer gelatin which is crosslinked using electron beam treatment and thereby usable for biomedical application. Its stimuli responsiveness can be appreciably tuned by varying material properties such as gel concentration and irradiation dose and can be extended to further environmental factors, including pH-value and salt concentration. Although, gelatin swelling in dependence on salt and pH was evaluated before^17,19^, it is investigated for the first time for electron treated gelatin.

Our investigations suggest that such a biocompatible demonstrator provides a wide range of applications such as bio-sensor or bio-actuator (e.g. self-expanding stents) in biomedicine. Furthermore, our demonstrated approach of electron-beam assisted programming of actuators can readily be realized in classical electron beam lithography systems, enabling realization of highly variable and complex actuators and actuator arrays from micro- to macro-scales. These will be the basis for complex stimuli-responsive shape changing elements that reversibly transform e.g. towards a programed shape by unfolding.

## Methods

### Gelatin sample preparation

The gelatin samples were prepared by using gelatin type A and B from porcine and bovine skin (G2500 and G9382, Sigma-Aldrich Chemie GmbH, Germany) with nominal gel strengths of 300 and 225 Bloom, respectively. The gelatin powders were dispersed in deionized water with concentrations of 4, 6, 8 and 10 wt%. The gelatin was allowed to swell for one hour. Afterwards, the mixture was heated up to 60 °C until it was liquefied and the gelatin was homogeneously dispersed. The solution was poured into cylindrical molds with a diameter of 12.6 mm and a length of approx. 5 mm. The gelatin polymerized for at least two hours at 8 °C. Before the samples were irradiated, they were purged with nitrogen and the molds were subsequently sealed to avoid contact with oxygen and undue dehydration.

### Electron irradiation

Electron beam treatment was performed using a 10 MeV linear electron accelerator (MB10–30MP; Mevex, Ontario, Canada). The accelerator is equipped with a moving stage (repetition rate of 180 Hz) and used an electron pulse with a length of 8 *μ*s. A scanning horn with a scanning frequency of 3 Hz scanned the electron beam over the sample. The final doses were obtained in steps of 5 kGy. The doses were measured with respect to a graphite dosimeter to an uncertainty of 10%. During electron beam treatment, the samples were airtight sealed within plastic bags and cooled using cooling packs to prevent exposure to oxygen and overheating, respectively.

### Swelling studies

After irradiation, the samples were dried over 3 days under an extractor hood at room temperature. The samples were subsequently immersed into deionized water, in which the desired acidic and basic pH-values and salt concentrations were obtained by adding 96% concentrated H_2_SO_4_, 0.1 M NaOH and NaCl, respectively. Sample masses were repeatedly determined as function of time with an error less than 0.1 mg until saturation and normalized to their dried mass prior to swelling. Saturation was always reached after swelling for 24 hours.

### Cantilever curvature measurement

After storing the gelatin bilayers at 8 °C, an USB 3 uEye ML camera (IDS Imaging Development Systems GmbH, Germany) was employed to record movies of the temporal evolution of the shape. Special focus was laid on the curvature change that was extracted with the help of the digital imaging processing software Fiji^20^ and the plugin *Three Point Circular ROI*.

## Electronic supplementary material

Supplementary Information

Video S3
