# Supplementary material for: Programing stimuli-responsiveness of gelatin with electron beams: basic effects and development of a hydration-controlled biocompatible demonstrator
Source: Sci Rep. 2017 Dec 12;7:17436. doi: 10.1038/s41598-017-17734-y (PMC5727221; doi:10.1038/s41598-017-17734-y)
Supplement: Supplementary file 1 — Supplementary Information [file 41598_2017_17734_MOESM1_ESM.pdf]

Supplementary Information

Programing stimuli-responsiveness of gelatin with electron beams: basic effects and development of a hydration-controlled biocompatible demonstrator

Stefanie Riedel[1,2,]*, Benedikt Heyart[1], Katharina S. Apel[1], and Stefan G. Mayr[1,2,]**

[1]Leibniz Institute of Surface Engineering (IOM), Leipzig, 04318, Germany
[2]Division of Surface Physics, Department of Physics and Earth Sciences, University of Leipzig, Leipzig 04103, Germany
*stefanie.riedel@iom-leipzig.de
**stefan.mayr@iom-leipzig.de

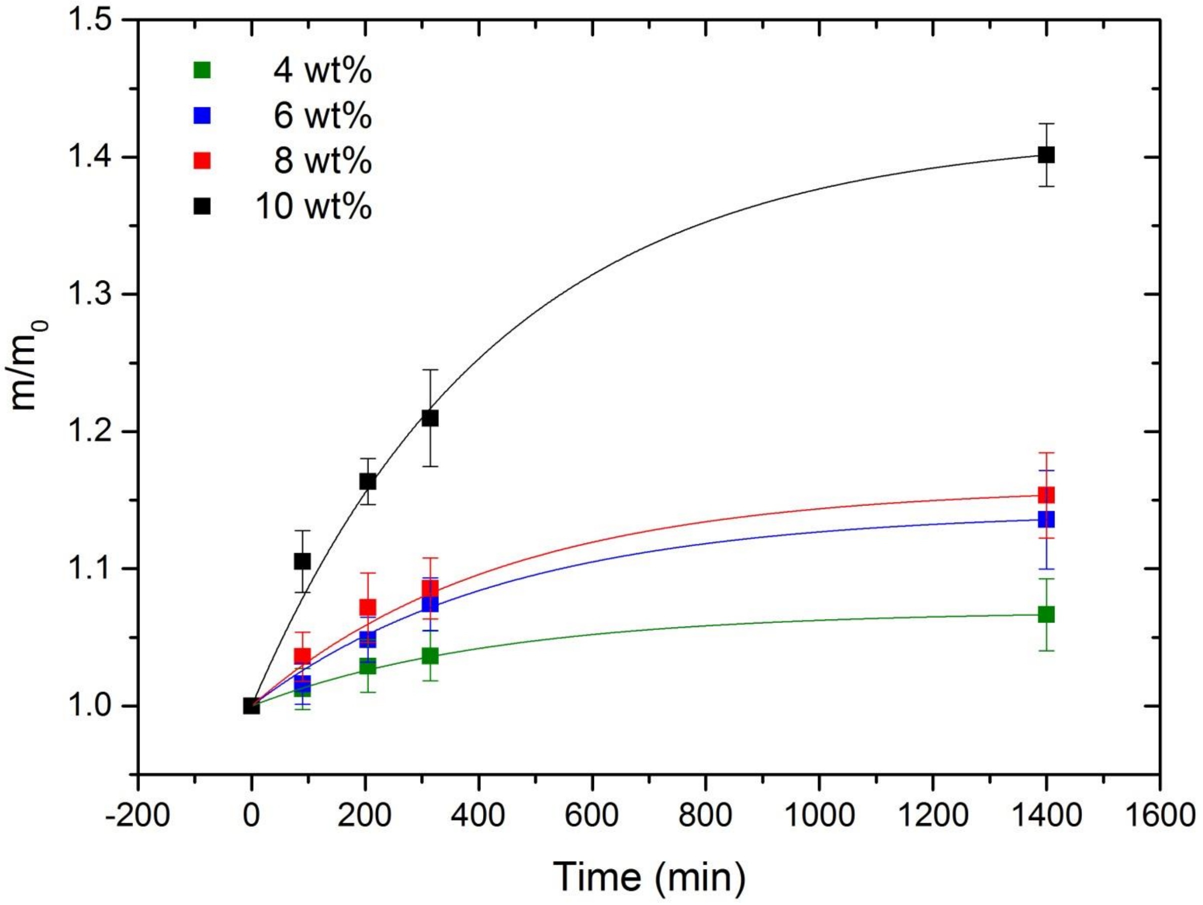

Figure S1. Time-resolved ratio of swollen weight m and weight after irradiation m_0 of gelatin samples with different gel concentrations irradiated with electron doses of 10 kGy. The samples were allowed to swell in deionized water until saturation. Data points and error bars indicate average of 12 samples and standard deviation, respectively.

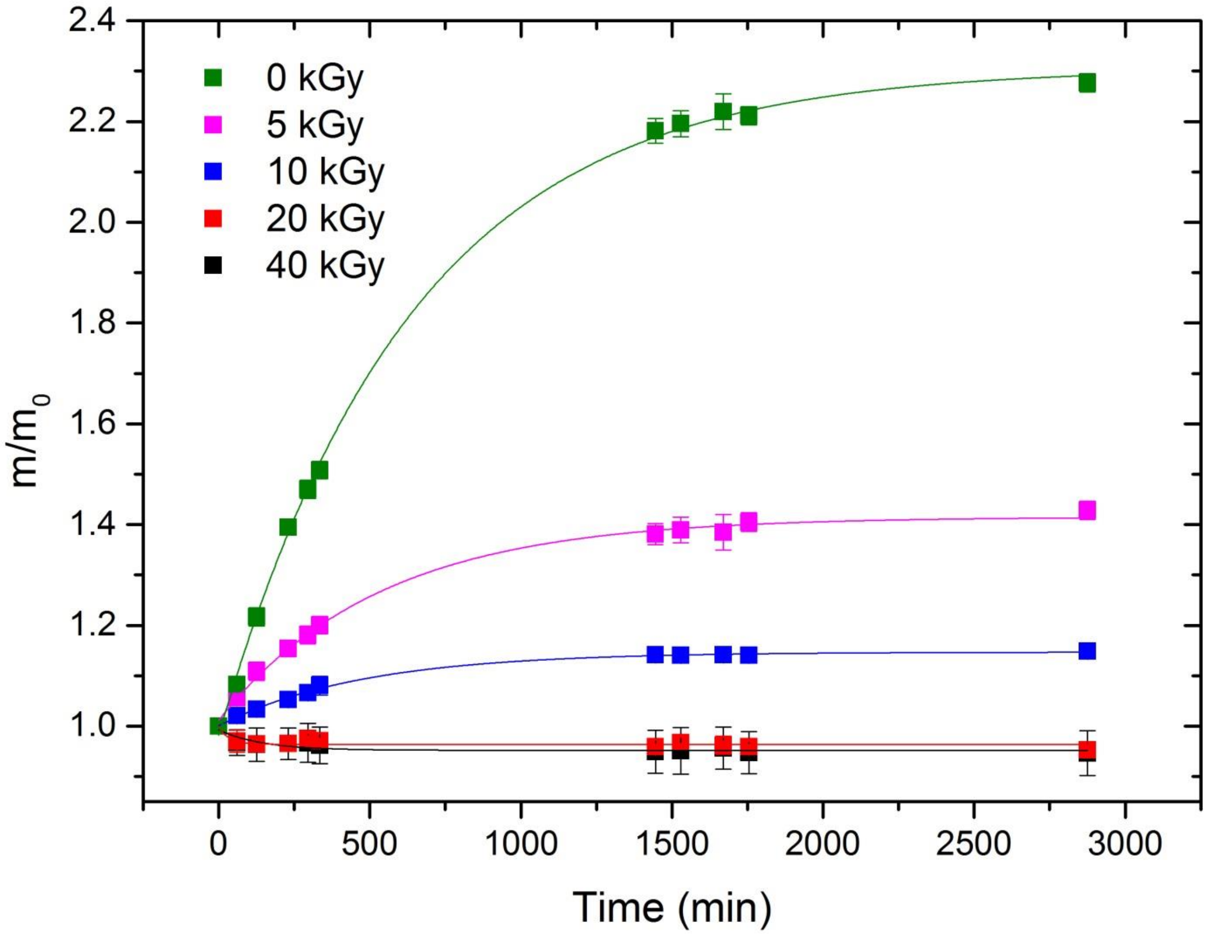

Figure S2. Time-resolved ratio of swollen weight m and irradiated weight m_0 of gelatin samples with a gelatin concentration of 8 wt% irradiated with different electron doses. The samples were allowed to swell in deionized water. Data points and error bars indicate average of 12 samples and standard deviation, respectively.

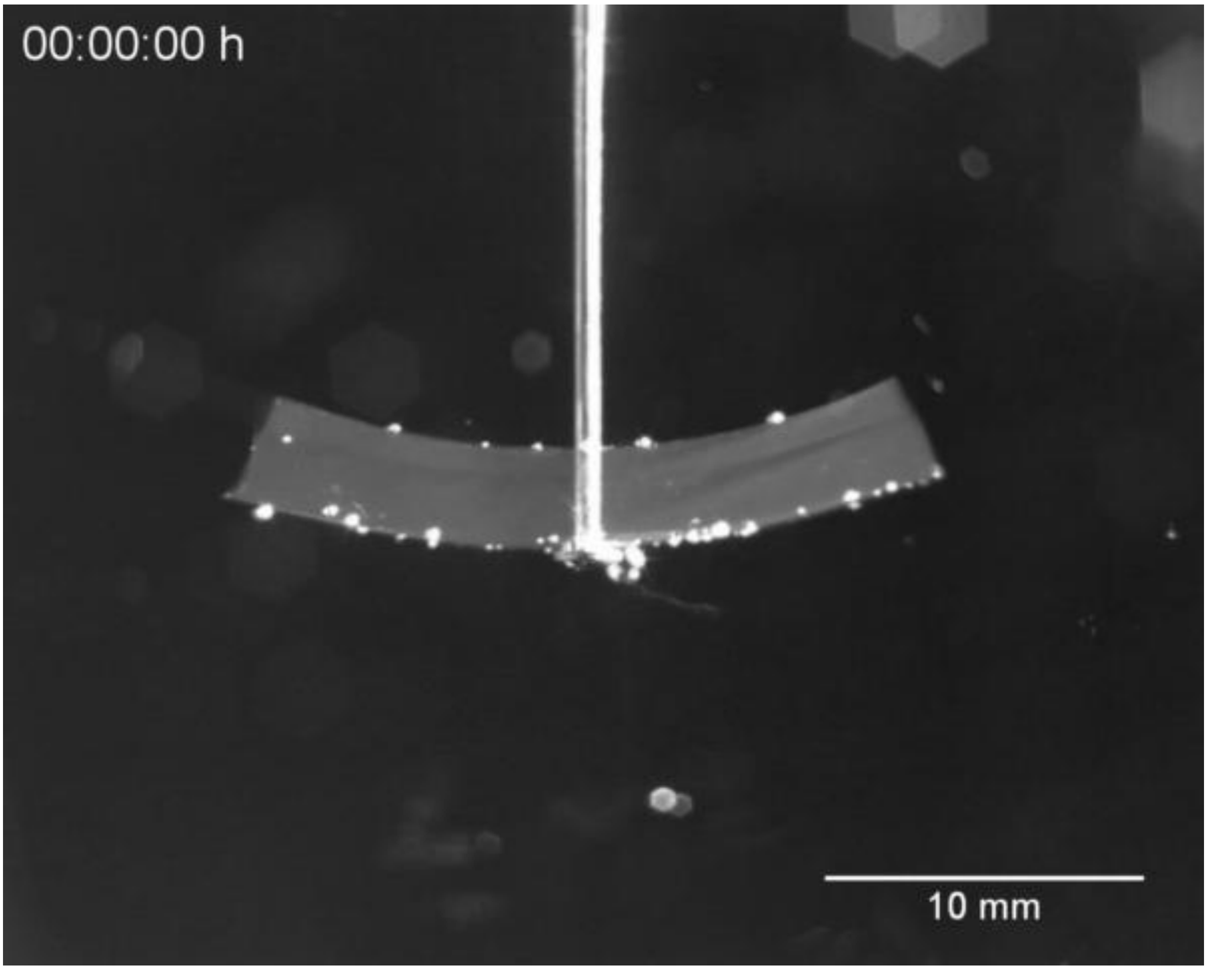

Video S3. Gelatin-bilayer shows bending deformation in contact with water: the top layer consists of 4 wt% gelatin irradiated with 40 kGy, the bottom layer consists of 10 wt% gelatin irradiated with 5 kGy. Differences in swelling ability cause deformation of the system. The video shows this deformation (looped four times).
